# Self-perceptions as mechanisms of achievement inequality: evidence across 70 countries

**DOI:** 10.1038/s41539-023-00211-9

**Published:** 2024-01-11

**Authors:** Sarah I. Hofer, Jörg-Henrik Heine, Sahba Besharati, Jason C. Yip, Frank Reinhold, Eddie Brummelman

**Affiliations:** 1 Faculty of Psychology and Educational Sciences, Ludwig-Maximilians-University Munich, Munich, Germany; 2 Gesellschaft für Weiterbildung und Sozialwissenschaftliche Forschung e.V. (GWSF) (Society for Further Education and Social Science Research e.V.), Munich, Germany; 3 Department of Psychology, University of the Witwatersrand (Wits), Johannesburg, South Africa; 4 The Information School and Human Centered Design & Engineering (affiliate), University of Washington, Seattle, WA USA; 5 Institute for Mathematics Education, University of Education Freiburg, Freiburg, Germany; 6 Research Institute of Child Development and Education, University of Amsterdam, Amsterdam, The Netherlands

**Keywords:** Education, Human behaviour

## Abstract

Children from lower socioeconomic status (SES) backgrounds tend to have more negative self-perceptions. More negative self-perceptions are often related to lower academic achievement. Linking these findings, we asked: Do children’s self-perceptions help explain socioeconomic disparities in academic achievement around the world? We addressed this question using data from the 2018 Programme for International Student Assessment (PISA) survey, including *n* = 520,729 records of 15-year-old students from 70 countries. We studied five self-perceptions (self-perceived competency, self-efficacy, growth mindset, sense of belonging, and fear of failure) and assessed academic achievement in terms of reading achievement. As predicted, across countries, children’s self-perceptions jointly and separately partially mediated the association between socioeconomic status and reading achievement, explaining additional 11% (Δ*R*^2^ = 0.105) of the variance in reading achievement. The positive mediation effect of self-perceived competency was more pronounced in countries with higher social mobility, indicating the importance of environments that “afford” the use of beneficial self-perceptions. While the results tentatively suggest self-perceptions, in general, to be an important lever to address inequality, interventions targeting self-perceived competency might be particularly effective in counteracting educational inequalities in countries with higher social mobility.

## Introduction

Achievement inequality is one of the most pressing social problems of our time. Around the world, children from lower socioeconomic status (SES) backgrounds underperform in school relative to their higher-SES peers^1–3^. SES is one of the strongest known predictors of academic achievement^4–6^. Research has begun to explore the psychological pathways from children’s SES to their academic achievement. Studies have, for example, identified cognitive stimulation (e.g., parents’ involvement in learning or learning resources at home) to mediate the relation between SES and language skills, which mediated the relation between SES and academic achievement 1,5 years later^7^. Others have explained the association between SES and academic achievement by cognitive flexibility and working memory^8^.

However, little is known about self-related psychological mechanisms underlying the association between SES and achievement. Here, we examined the role of children’s self-perceptions. Children from low-SES backgrounds tend to have more negative self-perceptions^9^. Negative self-perceptions are often related to worse academic achievement. Thus, children’s self-perceptions may serve as a mechanism of achievement inequality^9,10^. We tested this hypothesis using data from the 2018 Programme for International Student Assessment (PISA) survey, including 520,729 15-year-old students from a total of 70 countries. We also examined whether the role of self-perceptions in achievement inequality differs meaningfully across countries.

Why do children from lower-SES families show poorer academic achievement? Children from low-SES backgrounds face many structural barriers, such as poorer nutrition, lower-quality housing, less homework support from their parents, reduced access to educational materials in the home, and fewer extracurricular activities^11–14^. Due to these structural barriers, children from lower-SES backgrounds are not able to fully develop their academic ability. Even accounting for individual differences in ability, however, children’s SES is still strongly related to academic achievement^15^. One explanation is that children from lower-SES backgrounds face harmful societal ideas, which are then internalized as self-perceptions. Indeed, children from lower-SES backgrounds are often a target of negative intellectual stereotypes. Around the world, individuals from lower-SES backgrounds are seen as less competent^16^ and believed to be less able to develop their competence^17^ compared to their higher-SES peers. Unfortunately, children readily pick up on these harmful ideas and potentially internalize them as self-perceptions^9^. Consequently, children from lower-SES backgrounds may infer that they are less competent than others (i.e., low self-perceived competency and self-efficacy), and that there is little they can do to cultivate their competence (i.e., fixed mindset). More broadly, they may experience a fear of failure and a lack of belonging in educational contexts.

In line with recent theories of social-cognitive development^18^, we considered self-perceptions as a possible mechanism through which SES relates to children’s academic achievement^10^. *Self-perceptions* can be defined broadly as children’s mental representations and evaluations of themselves^19^. Self-perceptions often take the form of personal theories. Through their everyday experiences, children develop personal theories of who they are, what they are capable of, and how they are seen by others. Children derive hypotheses, gather data, weigh the evidence, and revise their theories accordingly^20,21^. Just like scientific theories guide scientific research, self-perceptions structure children’s everyday experiences, give them a meaning, and suggest ways of navigating them^22,23^. Given that children’s experiences differ based on their socioeconomic backgrounds, it is unsurprising that there are socioeconomic disparities in children’s self-perceptions, with downstream consequences for children’s academic achievement. As such, self-perceptions can be seen as “carriers of socialization and thus a means through which experience affects outcomes”^24^.

Previous research provides preliminary evidence for self-perceptions as mechanisms of achievement inequality^9^. Several studies suggest that *self-efficacy* (i.e., children’s belief about what they will be able to accomplish) and *self-perceived competency* (i.e., children’s subjective evaluation of their ability) can serve as such mechanisms. Across studies involving middle-school, high-school, and university students in different countries, those from lower-SES backgrounds were found to have lower self-perceived competency. Lower self-perceived competency, in turn, predicted poorer academic achievement^25–28^. This included broad perceptions of competence (i.e., self-efficacy) as well as perceptions of competence within specific domains, such as reading, mathematics, science, chemistry, and physics. In PISA 2015, students from lower-SES backgrounds had lower self-efficacy, and lower self-efficacy was related to poorer achievement in reading, mathematics, and science^3,29^.

Other studies show that *mindsets* (i.e., children’s belief that their abilities are fixed or can grow through hard work and dedication) can serve as mechanisms of achievement inequality. PISA 2018 likewise indicated an association between a growth mindset and higher scores in reading, mathematics, and science on average across OECD countries. Additionally, students from lower-SES backgrounds reported a growth mindset less often than more privileged students^30^. Similarly, in a large study of middle-school students in the United States, those from lower-SES backgrounds had more of a fixed mindset. A fixed mindset predicted poorer academic achievement^31,32^. Thus, preliminary evidence suggests that self-efficacy, self-perceived competency, and mindsets may partially explain socioeconomic disparities in academic achievement.

There is limited evidence for *sense of belonging*^33^ (i.e., the subjective feeling of a deep connection with people, things, or places) and *fear of failure* (i.e., persistent and irrational anxiety about failing) as mechanisms of achievement inequality. PISA 2015 showed that students from lower-SES backgrounds had a lower sense of belonging^3^. Similarly, in a study of university students, first-generation students (who tend to be from lower-SES backgrounds) experienced a reduced sense of belonging^34^, which could predict academic achievement^35^. Interventions targeting belonging have been found to successfully mitigate achievement inequalities for students from disadvantaged backgrounds^36,37^. Regarding fear of failure, some studies suggest that lower-SES students might be prone to fear of failure^38^, while others suggest the opposite^39^. Thus, it is less clear whether sense of belonging and fear of failure can be expected to serve as mechanisms of achievement inequality or not.

Research on self-perceptions and achievement inequality has focused predominantly on what has been termed as Western, Educated, Industrial, Rich, and Democratic (WEIRD) countries^40^. A critical and understudied question is how the role of self-perceptions in achievement inequality differs across a multitude of countries. Here, we consider cross-cultural differences in the associations between SES and self-perceptions as well as the associations between self-perceptions and academic achievement. We theorized that the strength and direction of these associations might be dependent on a country’s level of income inequality and social mobility.

First, we asked: How might country-level income inequality and social mobility predict the associations between SES and self-perception? One possibility is that SES is *more strongly* related to self-perceptions in countries with higher levels of income inequality and lower levels of social mobility. In countries with high income inequality, children might be exposed to higher levels of inequality in their everyday lives, which could inspire competition and increased comparisons. Indeed, students in countries with higher income inequality perceive their classmates as more competitive and are themselves more competitive^41^. Among children from lower-SES backgrounds, such competitive environments could elicit upward social comparisons that undercut their self-views^42–45^. Similarly, in countries with lower social mobility, the SES of children’s families is more strongly predictive of children’s future SES, meaning that children might see their family’s SES as a defining feature of who they are. Preliminary evidence supports these predictions. In PISA 2018, children from lower-SES backgrounds had more of a fixed mindset, especially in countries with higher achievement inequality^30^. In a study based on PISA 2000, 2003, and 2012, children from lower-SES backgrounds had a reduced sense of belonging, especially in countries with higher income inequality^46^.

Another possibility is that SES is *less strongly* related to self-perceptions in countries with higher levels of income inequality and lower levels of social mobility. Countries with higher income inequality and lower social mobility often experience socioeconomic segregation^47^. In such countries, children from lower-SES backgrounds may rarely interact with peers from higher-SES backgrounds, and vice versa^48^. Consequently, there might be fewer social comparisons based on their background, and children may not consider SES to be a defining feature of who they are.

Second, we asked: How might country-level income inequality and social mobility predict the associations between self-perception and achievement? Again, we proposed two opposing perspectives. One possibility is that self-perceptions are *more strongly* related to academic achievement in countries with lower income inequality and higher social mobility. The effects of self-perceptions might depend on characteristics of the context—that is, on its *psychological affordances*^49,50^. When children have certain self-perceptions (i.e., the seeds), they will behave in line with those self-perceptions only in environments that afford this behavior (i.e., a fertile soil in which these seeds can grow)^50^. For example, an environment that “affords” the use of growth mindsets would convey to children that they can improve themselves through effort and dedication^51^. Consistent with this reasoning, growth mindset is more strongly related to better academic achievement in countries with higher educational mobility (i.e., the percentage of children from low-education households to graduate from tertiary education)^52^.

Another possibility is that self-perceptions are *less strongly* related to academic achievement in countries with lower income inequality and higher social mobility. These countries have more supportive social structures, which may override the influence of individual effort and dedication. In more supportive and permeable systems—indicated by higher social mobility and lower income inequality—self-perceptions might become less important for achievement given the stronger impact of beneficial external conditions and sufficient resources.

### The present study

In this study, we investigated whether and how children’s self-perceptions may be related to socioeconomic disparities in academic achievement, indexed as reading achievement, across *n* = 70 countries based on data from the 2018 PISA survey (*n* = 57 countries for analyses including variables at the country-level). Previous research, including PISA, has shown associations between children’s SES, self-perceptions, and academic achievement. Building on and extending this work, we examined whether different self-perceptions mediate the association between SES and academic achievement; and whether and how these effects differ meaningfully across countries, focusing on income inequality and social mobility as country-level predictors.

To do so, we first looked at associations between children’s SES, self-perceptions, and achievement (RQ 1). Next, we tested whether children’s self-perceptions mediate socioeconomic disparities in achievement (RQ 2). Finally, we analyzed whether and how these psychological processes differ across countries depending on their level of income inequality and social mobility (RQ 3).

Although self-perceptions could help explain socioeconomic achievement gaps across achievement domains, we focused on the domain of reading, for three reasons. First, reading was the major domain of assessment of the most recent available cycle of PISA (2018), which means that the assessment included a comprehensive measure of children’s self-perceived competency in the domain of reading, but not in the other two domains (i.e., mathematics and science). We could not supplement our analyses with earlier PISA cycles, as these cycles do include other key variables, while scales measuring variables of interest for the present study are missing. Second, reading can be considered a key competency for success in life that impacts all other academic domains^53,54^. Third, there are stark socioeconomic disparities in reading^55–57^, making it critical to investigate their origins. Gaining a better understanding of the mechanisms underlying socioeconomic differences in achievement in this area is therefore particularly relevant.

Reading achievement refers to an individual’s ability to comprehend, use, and reflect on written texts in various contexts^29^. This includes the ability to understand and interpret the content and purpose of written materials, to identify and evaluate the arguments and claims made in those materials, and to draw connections and make inferences based on the information presented. Reading achievement also encompasses the capacity to engage with a variety of text types and genres, including narrative, informational, and argumentative texts, and to use reading as a tool for learning and personal development. In the PISA studies, reading achievement is assessed through a range of tasks and activities designed to measure these various components of reading proficiency, and is used as a key indicator of academic achievement.

We used a meta-analytic approach to examine our research questions. Since the standard PISA evaluation algorithms determine student-level variables with appropriately estimated standard errors per country based on independent samples in each country, meta-analytic procedures can be used to combine the results across countries, just as other meta-analyses combine the results of several independent studies or datasets. This approach was proposed by Brunner and colleagues in 2023^58^ to examine effect sizes from independent country samples, to combine them, and to investigate their heterogeneity. This paper puts the spotlight on similarities and differences between countries with regard to the mediation model from SES to reading achievement via self-perceptions—calling for an analysis method that explicitly allows for such country-specific estimation (for more information on the meta-analytic approach please also refer to the section “A Meta-Analytic Approach to analyze Large-Scale Data” in the “Methods”).

To correct for multiple testing, we adjusted the alpha level for all significance tests belonging to the same family of tests applying the conservative Bonferroni correction.

## Results

### Associations between SES, self-perceptions, and reading achievement

Our first question was how SES, self-perceptions, and reading achievement were associated across countries. We estimated the country-specific bivariate correlations between SES, the five self-perception variables (i.e., self-perceived competency, self-efficacy, growth mindset, sense of belonging, and fear of failure), and reading achievement. Afterwards, we performed a fixed (common) effects meta-analysis for each of the bivariate correlations to consider country-specific disparities when estimating a global mean (Table 1). The analyses showed that all bivariate correlations were positive and statistically significant—and did vary significantly between countries (as indicated by the *Q*-Statistic), suggesting explanatory country-level variables that are linked to country-specific disparities. Country-specific supplementary plots and tables with all statistical parameters are available via Open Science Framework (OSF; 10.17605/OSF.IO/SBFWH).

**Table 1:** Meta-analytic correlation coefficients between study variables.

	SES		Reading achievement	
	*r*^a^	*Q*^b^	*r*^a^	*Q*^b^
SES	–	–	0.339*	2116*
Self-efficacy	0.117*	741*	0.091*	1514*
Growth mindset	0.096*	1687*	0.195*	4840*
Fear of failure	0.019*	767*	0.057*	2157*
Sense of belonging	0.095*	497*	0.108*	2067*
Self-perceived competency	0.176*	1576*	0.319*	4449*

*p < 0.005; Bonferroni adjusted alpha (11 tests): α_adj._ = 0.005.

^a^Meta-analytically aggregated Pearson correlation.

^b^*Q*-Statistic indicating significance of variability of meta-analytically aggregated coefficients.

More specifically, we found a positive association between SES and reading achievement across countries (*r* = 0.339), demonstrating socioeconomic disparities in achievement. SES was significantly associated with self-perceived competency (*r* = 0.176), self-efficacy (*r* = 0.117), growth mindset (*r* = 0.096), sense of belonging (*r* = 0.095), and negligibly with fear of failure (*r* = 0.019). We found roughly the same pattern for the associations between self-perceptions and reading achievement: Self-perceived competency (*r* = 0.319), growth mindset (*r* = 0.195), sense of belonging (*r* = 0.108), self-efficacy (*r* = 0.091), and fear of failure (*r* = 0.057) were all significantly associated with reading achievement. Again, on the global level, the relation between students’ perceived fear of failure and their reading achievement may be considered negligible.

**Fig. 1:**
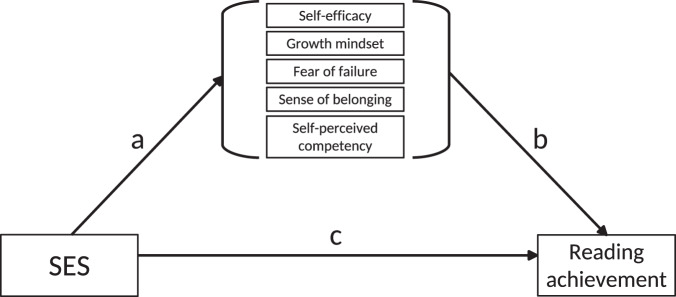
Multi-mediator model. Model applied to single PISA country data depicting the *a*-path, *b*-path, and *c*-path (direct effect). The *a* × *b*-path corresponds to the indirect effect.

These overall effects are informative for a global theoretical model in which SES is related to reading achievement through self-perceptions, detailed in Fig. 1. When focusing on specific countries, one should be aware of the bivariate correlation coefficients varying significantly between countries in all five self-perception measures (*Q*-Statistics, all *p*s < 0.005).

### Do self-perceptions mediate SES-disparities in reading achievement?

Our second question was whether self-perceptions mediated the association between SES and reading achievement across countries (see Fig. 1). To answer this question, we performed a meta-analytic multi-mediator analysis for the 70 countries. The Bonferroni adjusted alpha for all significance tests (19 tests) was α_adj._ = 0.003.

**Fig. 2:**
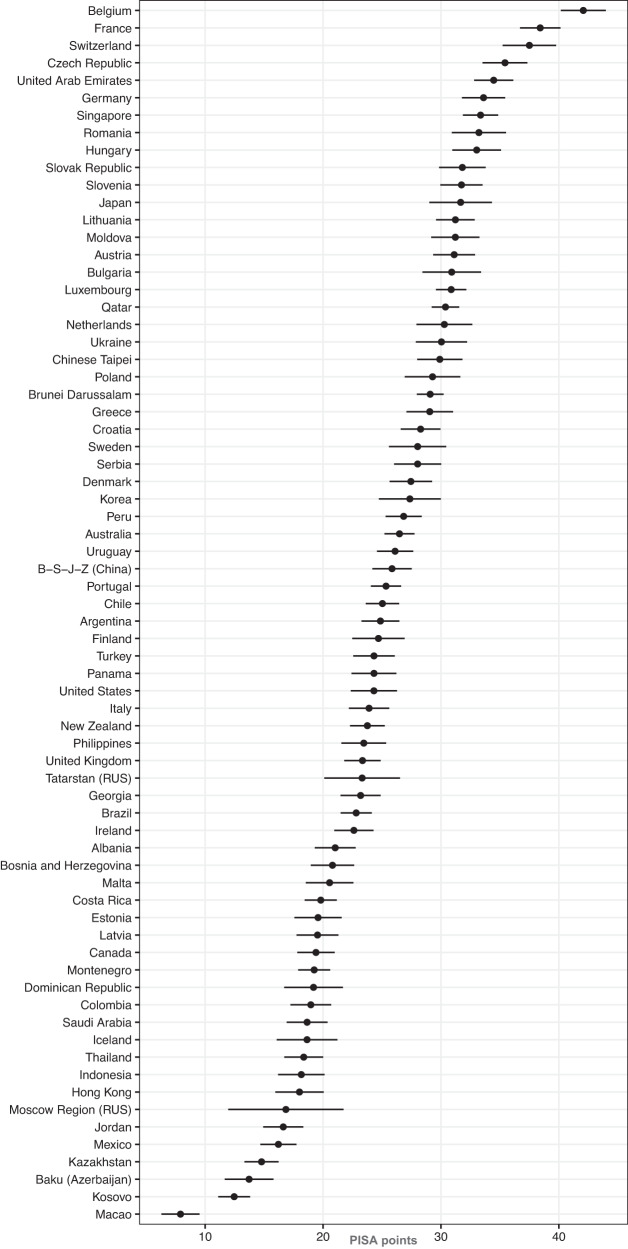
Multi-mediator model *c*-paths (reading achievement ~ SES). The criterion is reading achievement in PISA points. The multi-mediator model *c*-paths with 95% confidence intervals depict the association between reading achievement and SES across countries in PISA points (*M* = 500, SD = 100).

For reading achievement as dependent variable and SES as independent variable, the multi-mediator analysis showed a significant direct effect (*c*-path) of SES on reading achievement across countries (*β*_c, SMD_ = 0.257, *p* < 0.001, meta-analytically summarized over 70 countries, fixed effect model; Fig. 2). Overall, the standardized indirect effect (*a* × *b*-path) jointly considering the five self-perception variables was significant (*β*_*a*×*b*, SMD_ = 0.064, *p* < 0.001). For the full multi-mediator model, the explained variance was *R*^2^ = 0.220, which can be compared to the meta-analytically aggregated squared bivariate correlation between SES and reading achievement (*R*^2^ = 0.115), resulting in an improvement of Δ*R*^2^ = 0.105.

The standardized indirect effects of the single self-perception variables in the multi-mediator model were *β*_*a*×*b*, SMD_ = 0.003 for growth mindset, *β*_*a*×*b*, SMD_ = 0.001 for fear of failure, *β*_*a*×*b*, SMD_ = 0.003 for sense of belonging, *β*_*a*×*b*, SMD_ = 0.020 for self-perceived competency, and *β*_*a*×*b*, SMD_ = −0.002 for self-efficacy (all *p*s < 0.001). Similar to the bivariate correlations, fear of failure showed the weakest and self-perceived competency the strongest association. Importantly, in the multi-mediator model, the meta-analytically summarized coefficient for self-efficacy indicated a negative mediation effect. While for some countries, the mediation effect was positive, it was negative in other countries. Inspecting the two paths of the mediation (the *a*-path from SES to self-efficacy and the *b*-path from self-efficacy to reading achievement), it becomes clear that the negative mediation effect resulted from a negative association between self-efficacy and achievement (the *b*-path) in some countries.

**Fig. 3:**
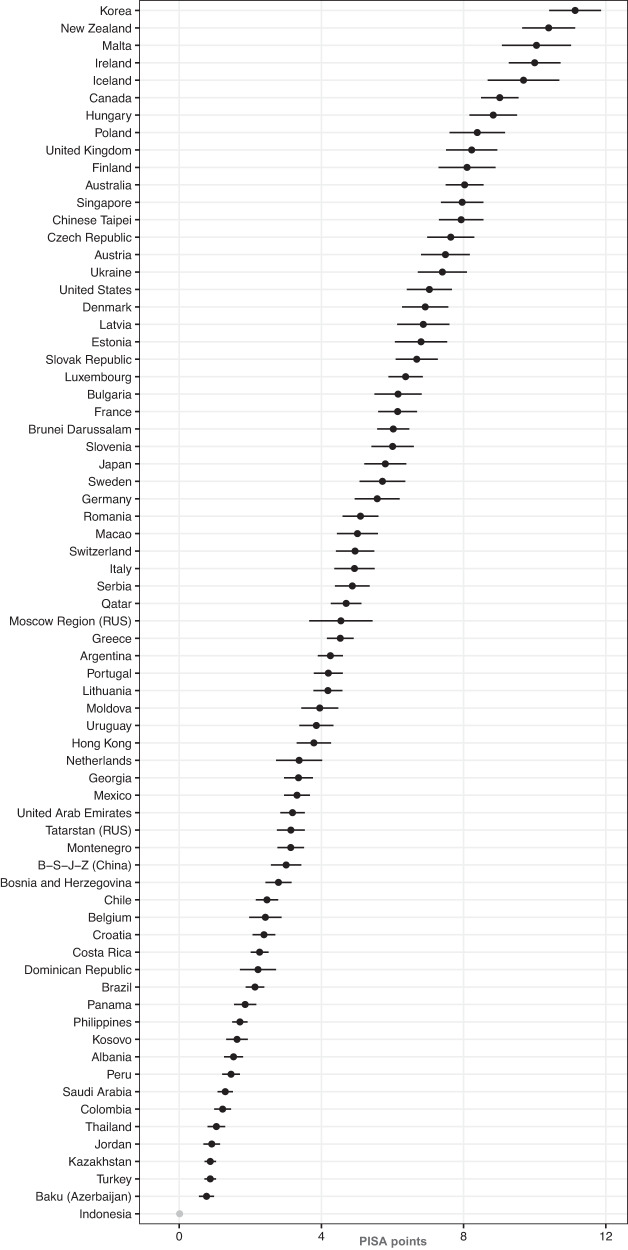
Multi-mediator model *a* × *b*-paths for mediator self-perceived competency. The criterion is reading achievement in PISA points. The multi-mediator model *a* × *b*-paths with 95% confidence intervals depict the association between reading achievement and SES mediated by self-perceived competency across countries in PISA points (*M* = 500, *SD* = 100).

All *a*- and *b*-paths were significant (all *p*s < 0.003; see OSF for more corresponding plots and tables). The coefficients varied significantly between countries (*Q*-Statistics, all *p*s < 0.001). Overall, our analysis suggests that self-perceptions mediate the association between SES and reading achievement, but not in the same way in all countries. Figure 3 visualizes how self-perceived competency, as an example, mediates the association between SES and reading achievement in the different countries.

### Prediction by country-level income inequality and social mobility

Our third question was whether we can explain part of the differences between countries in the associations between SES, self-perceptions, and reading achievement by the country-level predictors income inequality and social mobility. To better understand potential effects, we did not only look at the prediction of the indirect effects (i.e., the *a* × *b-*paths), but also at the prediction of the *a*- and *b*-paths. Accordingly, we used meta-analytic regression analyses to predict (i) the path coefficients from SES to the self-perception mediators (i.e., the *a*-paths), (ii) the path coefficients from the self-perception mediators to reading achievement (i.e., the *b*-paths), and (iii) the indirect effects (i.e., the *a* × *b*-paths) by income inequality (measured by the Gini index) and social mobility (measured by a global social mobility index). All parameter estimates and meta-analytic statistics can be found in the Tables 2, 3, and 4. The *I*^2^ statistic describes the proportion of total variation across countries that is due to heterogeneity rather than chance. If we were looking at effects not affected by sampling error (chance) and *I*^2^ was approaching zero, then this would indicate that there is almost no observed variance left, because almost all variation across countries is due to chance. If *I*^2^ was approaching one, then this would indicate that almost all of the observed variance was due to heterogeneity. *QM* is the omnibus test for predictors in the respective model. A significant *p*-value of *QM* indicates that the predictor included explains a significant proportion of the heterogeneity between countries.

**Table 2:** Ten metaregressions predicting respective *a*-paths (from SES to each mediator) by income inequality or social mobility (unstandardized).

	Income inequality					Social mobility				
	*Est.*	*SE*	*p*_*Est*_	*I*²	*QM*	*Est.*	*SE*	*p*_*Est*_	*I*²	*QM*
Sense of belonging	−0.002	0.005	0.701	82.46%	0.148	0.004	0.005	0.355	82.20%	0.857
Fear of failure	0.001	0.006	0.838	87.03%	0.042	0.012	0.005	0.019	85.80%	5.491
Self-efficacy	−0.013	0.007	0.045	89.83%	4.019	0.011	0.007	0.093	90.08%	2.822
Self-perceived competency	−0.019	0.009	0.032	95.53%	4.586	0.045*	0.007	<0.001	93.02%	38.511*
Growth mindset	0.008	0.007	0.281	93.70%	1.163	−0.012	0.007	0.281	93.56%	2.495

*p < 0.002; Bonferroni adjusted alpha (30 tests): α_adj._ = 0.002. Results of ten separate metaregressions for the *a*-path from SES to each mediator for each of the two country-level predictors across 57 countries; *Est*. = unstandardized coefficient; *SE* = standard error of estimate; *I*^2^ = percentage of total variation across countries that is due to heterogeneity rather than chance; *QM* = omnibus test for predictors (*df* = 55, for all tests); *p*-value for test of predictors (*QM*) equals *p*-value for unstandardized coefficients (regressions using single predictor).

**Table 3:** Ten metaregressions predicting respective *b*-paths (from each mediator to reading achievement) by income inequality or social mobility (unstandardized).

	Income inequality					Social mobility				
	*Est.*	*SE*	*p*_*Est*_	*I*²	*QM*	*Est.*	*SE*	*p*_*Est*_	*I*²	*QM*
Sense of belonging	0.361	0.660	0.584	91.45%	0.300	−3.093*	0.521	<0.001	86.09%	35.303*
Fear of failure	−0.998	0.644	0.123	91.56%	2.378	2.320*	0.583	<0.001	89.63%	15.836*
Self-efficacy	0.176	0.541	0.745	86.26%	0.106	−1.180	0.519	0.023	85.05%	5.163
Self-perceived competency	−2.232	1.034	0.031	96.30%	4.655	5.647*	0.777	<0.001	93.40%	52.800*
Growth mindset	1.591	0.981	0.105	96.01%	2.630	−2.100	0.966	0.030	95.88%	4.723

*p < 0.002; Bonferroni adjusted alpha (30 tests): α_adj._ = 0.002. Results of ten separate metaregressions for the *b*-path from each mediator to reading achievement for each of the two country-level predictors across 57 countries; *Est*. = unstandardized coefficient; *SE* = standard error of estimate; *I*^2^ = percentage of total variation across countries that is due to heterogeneity rather than chance; *QM* = omnibus test for predictors (*df* = 55, for all tests); *p*-value for test of predictors (*QM*) equals *p*-value for unstandardized coefficients (regressions using single predictor).

**Table 4:** Ten metaregressions predicting respective *a* × *b*-paths (i.e., indirect effect) by income inequality or social mobility (unstandardized).

	Income inequality					Social mobility				
	*Est.*	*SE*	*p*_*Est*_	*I*²	*QM*	*Est.*	*SE*	*p*_*Est*_	*I*²	*QM*
Sense of belonging	0.009	0.060	0.887	88.96%	0.020	−0.227*	0.051	<0.001	84.52%	19.657*
Fear of failure	−0.028	0.043	0.513	93.70%	0.429	0.067	0.043	0.123	93.42%	2.385
Self-efficacy	0.054	0.067	0.418	89.86%	0.655	−0.155	0.064	0.016	88.69%	5.864
Self-perceived competency	−0.871	0.361	0.016	98.71%	5.805	1.952*	0.274	<0.001	97.32%	50.738*
Growth mindset	0.240	0.187	0.198	98.73%	1.655	−0.415	0.182	0.022	98.62%	5.225

*p < 0.002; Bonferroni adjusted alpha (30 tests): α_adj._ = 0.002. Results of ten separate metaregressions for the *a* × *b*-path from SES to each mediator to reading achievement for each of the two country-level predictors across 57 countries; *Est*. = unstandardized coefficient; *SE* = standard error of estimate; *I*^2^ = percentage of total variation across countries that is due to heterogeneity rather than chance; *QM* = omnibus test for predictors (*df* = 55, for all tests); *p*-value for test of predictors (*QM*) equals *p*-value for unstandardized coefficients (regressions using single predictor).

**Fig. 4:**
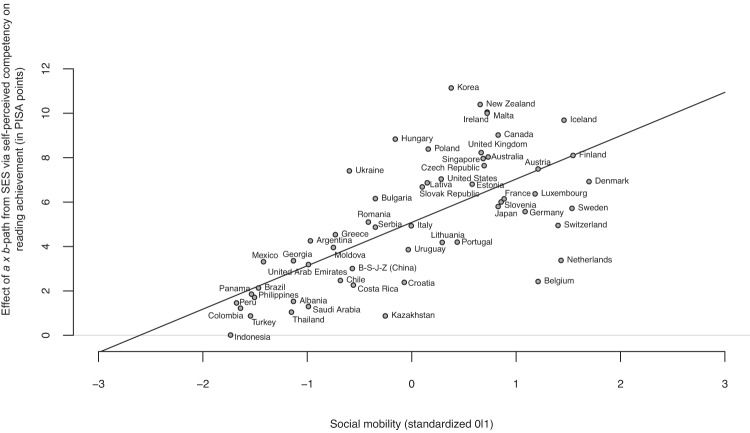
Prediction of *a × b*-paths for mediator self-perceived competency by social mobility index. The indirect effect (path *a × b*) via self-perceived reading competency is predicted by countries’ social mobility index using a meta-analytic approach across *n* = 57 countries; y-axis expresses effect in PISA points (*M* = 500, SD = 100).

Income inequality was no significant predictor of any of the paths *a*, *b*, or *a × b* (Tables 2–4). However, we found that the social mobility index significantly predicted the association of SES with self-perceived competency (*b* = 0.045); in countries with higher social mobility, the association of SES with self-perceived competency was stronger (Table 2). Moreover, the social mobility index significantly predicted the association of three of the five self-perception variables with reading achievement (Table 3). We found a significant negative association with sense of belonging (*b* = −3.093). In countries with higher social mobility, the association of sense of belonging with achievement was weaker. In addition, we found significant positive associations for fear of failure (*b* = 2.320) and self-perceived competency (*b* = 5.647). In countries with higher social mobility, the associations of fear of failure and self-perceived competency with reading achievement were stronger. Finally, as shown in Table 4, the social mobility index significantly predicted the indirect effects of SES on reading achievement via self-perceived competency (*b* = 1.952; Fig. 4) and sense of belonging (*b* = −0.227). The significant prediction of the indirect effect (*a* × *b*-paths) via sense of belonging, however, can mainly be attributed to social mobility predicting the *b*-path (Table 3), given that the coefficient for social mobility predicting the *a*-path was not significant (Table 2). Accordingly, we abstain from further interpreting the prediction of the *a* × *b*-path via sense of belonging by social mobility and only interpret the prediction of the *a*-path. In contrast, social mobility predicted both the *a*-, *b*- and *a × b*-paths via self-perceived competency.

## Discussion

Can self-perceptions serve as mechanisms of achievement inequality? We addressed this question by analyzing data from the 2018 PISA survey, including 520,729 students across 70 countries. We studied five dimensions of children’s self-perceptions: self-perceived competency, self-efficacy, growth mindset, sense of belonging, and fear of failure. As predicted, across countries, children’s self-perceptions partially mediated the association between SES and academic achievement, indexed as reading achievement. The mediating effect of self-perceived competency between SES and reading achievement was stronger in countries with higher social mobility, indicating the importance of environments that “afford” the use of beneficial self-perceptions.

Our findings substantiate the idea that children’s socioeconomic background is reflected in their self-perceptions^9,59–62^ and confirm the importance of these self-perceptions for reading achievement^63,64^. Although all self-perception variables were associated with achievement^26,27,32,35^, this bivariate association was strongest for self-perceived competency and almost negligible for fear of failure. Self-perceived reading competency is the self-perception variable most directly related to academic achievement in general and performance in the domain of reading in particular. The domain-specificity of this variable^65^ can explain the especially strong association with reading achievement in our analyses. The weak association with fear of failure, however, requires a more complex interpretation. We consider fear of failure as “persistent and irrational anxiety about failing to measure up to the standards and goals set by oneself or others”^66^. While this sometimes seems to impair academic outcomes^38^, it has also been found to contribute to an increase in performance^67^. Children may respond differently to fear of failure. For example, some children may try to avoid failure by succeeding and striving, whereas others may deal with their fear through counterproductive activity aimed primarily at self-protection, such as avoidance and self-handicapping^68^. The measure used in this study does not distinguish between those contradictory reactions to fear of failure, which may have obscured actual associations with SES and reading achievement. Future research might try to identify systematic differences in self-perception that underlie those divergent reactions to fear of failure.

Our findings suggest that self-perceptions, considered both jointly and separately, partially mediate the association between SES and reading achievement. Despite the mediation, the direct effect of SES on achievement was still significant and larger compared to the indirect effect. This shows that self-perceptions are not the only (or the most important) mechanism of achievement inequality. Moreover, the mediation effect differed significantly across countries. Self-perceived competency was the strongest mediator variable (in line with the discussion in the previous section), whereas self-efficacy turned out to be the only slightly negative mediator variable (with a positive mediation in some countries and a negative mediation in others), resulting from a negative association between self-efficacy and achievement (the *b*-path) in some countries. Some scholars have emphasized that too much self-efficacy can be troubling for individuals^69^. Extreme self-efficacy can drive learners to become overly persistent, even to unattainable goals, and make them overly tolerant of adversity (i.e., the “false hope syndrome”)^70^. Future research might identify country-level characteristics that distinguish between countries where self-efficacy and achievement are positively or negatively associated.

One limitation of our work is its reliance on correlational data. We agree with scholars who caution against using correlational data for testing mediation^71,72^. Yet, correlational data can be used for this purpose if the hypothesized mediation model is based on rigorous theoretical considerations^73^. We provided such considerations for why we expected self-perceptions to mediate the association between SES and achievement. SES cannot be manipulated experimentally, so only correlational methods can be used to test its effects on self-perceptions and achievement. Ideally, research would assess children’s SES and track its effects on self-perceptions and achievement longitudinally across many countries simultaneously, and then experimentally manipulate self-perceptions to test their causal effects on academic achievement. Unfortunately, such research would be extremely resource- and time-intensive and is currently unavailable. Although cross-sectional, the PISA 2018 data provides the best opportunity to test our hypothesized mediation models across countries, as they include children’s SES, self-perceptions, and achievement in a sample of over 500,000 students across 70 countries, providing an unprecedented level of statistical power.

Because our data are correlational, they do not inform causality. Our results suggest that self-perceptions mediate the association between SES and reading achievement. Yet, without ruling out confounding variables and reverse causality, we cannot draw any causal conclusions^71,72^. In terms of reverse causality, reading achievement might influence self-perceptions (rather than self-perceptions influencing achievement), as skill-development models would suggest. However, most researchers currently favor reciprocal effects models that acknowledge mutual influences^74^. Since we cannot disentangle reciprocal effects in the current study, we have to conceive of our *b*-path estimates as upper limits that might contain effects from achievement to self-perception variables in addition.

Family SES is an exogenous variable, so it is unlikely to be influenced by self-perceptions and reading achievement. Yet, SES can influence psychological processes that are related to but distinct from self-perceptions, which in turn influence reading achievement. Thus, our results do not rule out the possibility that our mediation effects are explained by other confounding variables. To rule out confounding variables and reverse causality, we call for longitudinal research (i.e., to examine whether SES predicts changes in self-perceptions and achievement) and experimental research (i.e., to examine whether changing students’ self-perceptions severs the association between SES and achievement).

We further sought to better understand how the associations between SES, self-perceptions, and achievement differ across countries. Accordingly, we examined whether income inequality and social mobility could predict the *a*-paths, the *b*-paths, and the *a × b*-paths.

We had two opposing hypotheses about how the association between SES and self-perceptions (*a*-path) would be predicted by country-level income inequality and social mobility. Our results provided some evidence for the hypothesis that the association is weaker in countries with lower levels of social mobility, presumably due to segregation and fewer opportunities for social comparison. This was found for self-perceived competency.

Similarly, we had two opposing hypotheses about how the association between self-perceptions and reading achievement (*b*-path) would be predicted by country-level income inequality and social mobility. We found partial support for both perspectives. The positive mediation effect of self-perceived competency was more pronounced in countries with higher social mobility, indicating the importance of environments that “afford” the use of beneficial self-perceptions. Fear of failure was also more positively related to achievement in countries with higher social mobility. Following the same line of argumentation, an environment that offers more opportunities to evolve, might also “afford” more opportunities to fail. To avoid failure, children with higher fear of failure might hence also feel more pressure to try harder.

Sense of belonging, however, turned out to be more strongly related to achievement in countries with lower social mobility, suggesting that this self-perception might become more important for achievement under less supportive external conditions. We used a comprehensive measure of social mobility reflecting multiple intergenerational outcomes (e.g., health, working conditions, technology access). These outcomes represent the country-level resources available to individuals to exploit their potential. Certain self-perceptions, such as sense of belonging, indeed seem to become less consequential for achievement when external resources are abundant. Conversely, children’s sense of belonging becomes more consequential for achievement when such external resources are scarce. The belief that one belongs socially, even in less supportive and less permeable environments, might provide the motivation needed to make the most of the learning opportunities offered^75^. This interpretation may not hold for narrower measures of social mobility, such as those focusing on a specific aspect of educational equity at the country-level (e.g., educational mobility as implemented in ref. ^52^).

The results for self-perceived reading competency pointed in the same direction for both its relation to SES and reading achievement (significant *a*-path, *b*-path, and *a × b*-path). Consequently, self-perceived reading competency seems to be an important lever to address inequality, especially in countries with higher social mobility. In such countries, children’s SES may be more consequential for children’s self-perceived reading competency. At the same time, these countries may provide fertile soil for educational growth and the development of individual potential^50^. Under those conditions, a strong belief in their own domain-specific abilities seem to drive children to higher levels of performance and motivate them to take advantage of the resources they are offered^76^. Our results tentatively suggest that interventions targeting self-perceived competency^77^ might hence be particularly effective in counteracting educational inequalities in countries with higher social mobility. In the present study, we provide evidence for this mechanism in the domain of reading. However, based on the broad literature on self-perceived competency and its relation to performance in different domains^78^, we would expect similar results in other domains as well.

Finally, income inequality turned out to be no significant country-level predictor of the *a*-paths, the *b*-paths, and the *a × b*-paths. This is in line with a recent study based on PISA data, showing that income inequality measured by the Gini index was not significantly related to achievement^79^. The level of social mobility in a country might hence be a more influential external condition affecting how self-perceptions are related to achievement inequality.

In recent literature, self-perceptions in youth have received some attention for promoting deficit perspectives of individuals^80–82^. Deficit perspectives locate the responsibility of problems within individuals. Deficit models are guided by research that focuses on children and adolescents who fail to meet predetermined standards for an isolated skill or characteristic^83^. While we have some evidence that self-perceptions might partially mediate the association between SES and achievement, we do not ascribe to deficit models of youth that attribute individual lack as the factor for achievement. Future work should move beyond a deficit model, for example, by using qualitative methods and innovative quantitative (e.g., person-centered) analyses to discover the unique strengths of children from disadvantaged backgrounds^84^. Such work has the potential to move educators beyond the deficit perspective that pervades much of education research in general. Similarly, we theorize that simply changing lower-SES children’s self-perceptions, without addressing the multitude of structural barriers that gave rise to them, may not be effective and may inadvertently convey that the children are themselves to blame for their predicament^82^.

Our study has several strengths, including its examination of different self-perceptions as mechanisms of inequality across the globe, its meta-analytic technique to model cross-country effects and heterogeneity, and its investigation of both social mobility and income inequality as potential country-level predictors. Despite the robust results presented here, our study has some limitations. First, there are still intrinsic limitations to using survey-based and self-report measures, such as demand characteristics, response biases, test effort, and fatigue^85^. Second, PISA is also cross-sectional in nature, only looking at data in 15-year-olds, while socioeconomic disparities in self-views may emerge at a younger age. Future studies should use longitudinal and experimental methods in younger children. Such studies could substantiate the mediation findings of the present study. Third, the self-perception variables and measures were also limited to what was available in the PISA student questionnaire. Future studies need to draw on both experimental and qualitative methods to further unpack the relationships between self-perceptions and achievement inequality found here. More broadly, an important limitation of PISA is that it includes no country from Africa. Consequently, all countries from the African region were excluded from our global analyses. Future studies could make use of cross-sectional and longitudinal datasets specific to Africa^86^ to compare the results of this study.

To conclude, in this study, we investigated children’s self-perceptions as mechanisms of achievement inequality on a global scale. Our findings suggested self-perceptions as one potential mechanism mediating the relationship between socioeconomic status and reading achievement. This relationship with self-perceptions also differed meaningfully across countries, partly depending on country-level social mobility. Longitudinal, experimental, and qualitative research is needed to substantiate the findings and uncover the role of self-perceptions in achievement inequality, so as to inform interventions at both a micro level (e.g., schools and families) and macro level (e.g., education policies).

## Methods

### Database

We used data from the international 2018 PISA survey which is publicly available from the OECD download pages as the source to build up the database for the present study^87^. This study is a secondary data analysis of publicly available data (PISA 2018) and hence does not require ethical approval. It involved no primary data collection. However, PISA 2018 data collection procedures indicate that respective parties provided informed consent.

For the initial data preparation steps, we followed the guidelines for processing and analyzing the PISA databases provided by OECD^88^. To analyze and compute our models, we used derived and Item Response Theory (IRT)-scaled outcome variables which were assessed on the student-level. In addition, we included two measures which were available on the country-level outside of the PISA databases. In the following sections, we give a brief overview of all of these variables.

### PISA measures on student-level

We used the *economic, cultural and social status* index (ESCS) to operationalize possible social disparities that are induced via the parental home. The ESCS is a measure of both socioeconomic and cultural resources of the parental home. Three source variables go into the construction of this index: socioeconomic status, parental educational attainment, and a measure of possession of cultural and wealth assets^89^. Compared to other variables measuring socioeconomic status like the *highest socioeconomic status* (HISE) or the *index for home possessions* (HOMEPOS), the ESCS is the variable that is used most frequently in reports and secondary analyses of PISA data^90^. In the OECD PISA datasets, ESCS is included as a standardized value, so the OECD mean is *M* = 0 with a standard deviation of *SD* = 1.

An assessment was conducted independently by five raters on the student PISA questionnaire 2018 (https://www.oecd.org/pisa/data/2018database/) to identify questions related to self-perception. Consequently, five variables were identified from PISA concepts related to self-perception based on the above operationalization of self-perception, specifically: (1) self-perceived competency; (2) self-efficacy; (3) growth mindset; (4) fear of failure; and (5) sense of belonging. The scales achieve satisfactory reliabilities across the countries participating in PISA (see Table 16 in ref. ^91^) that meet the technical standards of PISA. These five self-perception variables used for subsequent analysis are described in detail below.

Self-perceived competency: The 2018 student PISA questionnaire focused on reading achievement. Therefore, self-perception of academic competence is related specifically to reading. Three, single statement questions were used to assess self-perceived reading competency (e.g., “I am a good reader.”, “I am able to understand difficult text.”). Each of the questions were scored using a 4-point Likert-type scale ranging from “Strongly disagree”, “Disagree”, to “Agree”, and “Strongly agree”. Scores on these three questions were combined by means of applying the IRT-scaling model^91^ to create the index of self-perceived competency as a self-perception measure.

Self-efficacy: Self-efficacy, also referred to as resilience in the PISA student questionnaire, was assessed using five, single statement questions regarding the students’ belief in their ability related to difficult aspects or situations about themselves or school life. Examples of questions include: “I usually manage one way or another.”, “My belief in myself helps me through difficult times.” and “When I am in a difficult situation, I usually find a way out of it.”. Questions were scored using the same 4-point Likert-type scale presented above. Scores on these five questions were then combined by means of applying the IRT-scaling model^91^ to create the index of self-efficacy.

Growth mindset: To assess self-perception regarding the changeability of individual characteristics, a single question from the PISA student questionnaire was used. This single question item has been used in previous studies as a validated measure of growth mindset^92^. Participants were asked to rate to what extent they agreed with the following statement: “Your intelligence is something about you that you can’t change very much”, using the same 4-point Likert-type scale from “Strongly disagree” to “Strongly agree”. Responses were reversed scored to keep to the same directionality of the other self-perception variables. Therefore, using the inverse scores, the higher scores indicated greater growth mindset and lower scores corresponded more to fixed mindset.

Fear of failure: Fear of failure as a self-perception variable was assessed using three, single questions from the 2018 PISA student questionnaire (e.g., “When I am failing, I worry about what others think of me.”). Responses to the three self-report questions were measured using the same 4-point Likert-type scale described above. Therefore, higher scores indicate a greater sense of fear of failure. Scores were combined by applying the IRT-scaling model^91^ to create the index of fear of failure.

Sense of belonging: The final self-perception variable assessed students’ perception of sense of belonging at school. Participants were asked to indicate to what extent they agreed to six statements when thinking about their school (e.g., “I feel like I belong at school.”, “I make friends easily at school.”). These statements were similarly rated using a 4-point Likert-type scale ranging from “Strongly agree” to “Strongly disagree”. The scores were combined by means of applying the IRT-scaling model^91^ to create the index of sense of belonging.

In the following, we describe the measurement of reading achievement. In the PISA 2018 assessment, reading was the main domain. Reading was thereby recorded as a construct composed of several sub-dimensions, which are closely correlated and thus contribute to one overarching competence domain of reading achievement^93^. Since we wanted to use an overarching competence domain along our research questions, we resorted to this overarching measure of reading achievement for our analyses. In the data, this measure is in the form of ten plausible values (PVs), which consider the measurement inaccuracy at student-level, such as that resulting from the use of the rotated booklet design and other specifics of the PISA assessment design—for a detailed presentation of these methodological aspects, see^94–96^. These ten PVs are the result of scaling students’ responses to single reading tasks from the computer-based assessment using models based on IRT; see e.g. refs. ^94–96^. The logit metric for students’ scores initially resulting from the IRT-scaling is subjected to transformation for each PISA-survey round using specific transformation parameters, resulting in the OECD mean score of *M* = 500 with a standard deviation of *SD* = 100 (see ref. ^93^, p. 53 ff.). The scores achieved in reading by single countries or groups of students are related to this typical PISA metric, which is also referred to as “PISA points”.

### Measures on country-level

For the country-level analyses, we relied on two variables that are available outside the PISA 2018 data. To measure income inequality, we used the Gini index provided by the World Income Inequality Database (WIID), and for social mobility, we used data presented in the Global Social Mobility Report 2020, which was published by the World Economic Forum. For our analyses at the country-level, we combined these two indices with our results based on PISA data for 57 countries. The number of countries was reduced because the two country-level indices were only available for 57 of the countries participating in PISA. In the following sections, we discuss the two indices in detail.

The Gini index measures the deviation of actual incomes from a perfectly equal distribution. It was developed by the Italian statistician and sociologist Corrado Gini (1884-1965) to quantify the distribution of income. If incomes are equal for all individuals, the Gini coefficient takes the value 0. If a single individual earns the entire income of an economy, the Gini coefficient is 1. The data for the Gini index for our present analysis was extracted using the World Income Inequality Database (WIID) as a measure of income inequality (UNU-WIDER, 2022). WIID is a widely used, comprehensive, and open-access resource that compiles information on income inequality across 201 countries^97^. We used data available for 2018 (or the year with available data closest to 2018, respectively) to make it comparable to the 2018 PISA survey used in this study.

The general concept of social mobility was coined by the Russian-American sociologist Pitirim Sorokin (1927) and refers to the ability of a person to move in social space^98^ and to change into a different social class (compared to their parents). Typically, current literature focuses on the possibility of moving from a lower to a higher social class, which, as a measure for a social system, is referred to as social permeability. As a country-level measure, we used the social mobility index data from the Global Social Mobility Report 2020^99^. The general principle of this composite index is to combine key factors that currently influence future social mobility in different economies and societies. Accordingly, this social mobility index reflects a multifaceted concept that covers complex social shifts in life circumstances spanning different generations. The Global Social Mobility Report not only considers traditional outcomes of differences in income between children and parents but uses a unique conceptual framework that incorporates multiple intergenerational outcomes related to health, working conditions, and technology access, for instance. To allow for the aggregation of different indicators, each indicator entering the Global Social Mobility Index was converted into a so-called “progress score” which is unitless and ranges from 0 to 100.

### A meta-analytic approach to analyze large-scale data

To answer our research questions, we followed a meta-analytic integrative approach to analyzing international large-scale assessments (LSA) data comprising records from multiple countries^58,100^. Before we describe the procedure, we want to elucidate the fields of application of a meta-analytic approach.

To further clarify the fields of application of a meta-analytic approach to analyze large-scale data such as PISA data, we compare it with multilevel modeling. Multilevel modeling is especially suited to answer research questions that address cross-level effects (i.e., effects of variables on one level on variables on different levels) including cross-level interactions (e.g., the effect of an interaction between school-level variable A and country-level variable B on student-level variable C)^41,46^. In the present paper, we investigate associations between children’s SES, self-perceptions, and achievement and the mediation of socioeconomic disparities in achievement via children’s self-perceptions across countries. Both research aims do not involve cross-level effects or interactions but require combining data on the same level from independent samples from several countries, which can be done efficiently within a meta-analytic framework. We also examine how the meta-analytically derived coefficients for the mediation paths differ across countries depending on the countries’ level of income inequality and social mobility. Yet, we are not looking at cross-level interactions either. Instead, we aim to explain differences between countries in the coefficients of the mediation model (the *a*-, *b*-, and *a* × *b*-paths). Consistently following a meta-analytic approach for all research questions allows us to predict these coefficients by the countries’ income inequality and social mobility using simple metaregressions for each country-level predictor. This last research question hence aims at a prediction of the mediation paths and thereby at an explanation for differences in those coefficients across countries. We use the meta-analytic approach to examine and combine effects (coefficients) from independent country samples and explain their heterogeneity. Accordingly, while both approaches – multilevel modeling and the meta-analytic approach – can be validly applied to model large-scale data like PISA data, they might be especially well suited to answer slightly different kinds of research questions.

In the following paragraphs, we describe the analysis procedure. In a first step, our analysis model was applied separately to each partial data set for the single countries. In a second step, these separate results for the countries were combined into an overall result within the framework of a meta-analytical approach. On the one hand, this approach considers the independent sampling of the LSA PISA data in the respective countries. On the other hand, differential effects of our models between the respective countries can be analyzed more thoroughly.

Following the meta-analytic approach outlined above, in the first step, we calculated bivariate correlations between all variables considered in our analysis based on data from each single country. We also applied a multi-mediator model to predict PISA reading achievement for each single country independently. The model included all five self-perception variables as potential mediators. All our analyses took into account the stratified sampling structure (students in schools) for every single country. For this purpose, we used the respective student weights available in the data for each case and the corresponding replicate weights, following the analysis guidelines provided by^88^. Although PISA results are officially reported for 79 countries^93^, at least some of our selected study variables were not available in nine countries, reducing our data set to 70 countries. This is because part of the assessment in PISA is optional and not implemented in all countries. All of these analyses were performed in the free statistical environment *R*^101^ using the package “BIFIsurvey”^102^.

In the second step, we summarized all country-specific results of our initial analyses (the first step), e.g., correlation and path coefficients, following a meta-analytic approach. Specifically, we calculated the meta-analytic estimates for correlation coefficients considering the variance among the coefficients from the single country analyses. Moreover, we conducted significance tests on the single model coefficients (e.g., the direct and indirect paths). The same principle of meta-analysis was applied to all model coefficients from the multi-mediator model.

To investigate the effects of the two country-level predictors, we conducted metaregressions predicting the respective model coefficients (the *a*-paths, the *b*-paths, and the *a* × *b*-paths), for each of the five self-perception variables separately, by the countries’ social mobility index or the Gini index. For all of the meta-analytic analyses, we used the *R* package “meta”^103^.

We corrected for multiple testing. Accordingly, the alpha level for all significance tests belonging to the same family of tests was adjusted applying the conservative Bonferroni correction.

### Reporting summary

Further information on research design is available in the Nature Research Reporting Summary linked to this article.

### Supplementary information

Reporting summary

## Data Availability

We used data from the international 2018 PISA survey which is publicly available from the OECD download pages as the source to build the database for the present study^87^ (https://www.oecd.org/pisa/data/).

## Code availability

Our data-analytic code and supplementary plots and tables with all statistical parameters are available via Open Science Framework (OSF): 10.17605/OSF.IO/SBFWH.
